# Role of Biomarkers in the Diagnosis of Anastomotic Leakage After Colorectal Surgery: A Systematic Review and Meta-Analysis

**DOI:** 10.7759/cureus.62432

**Published:** 2024-06-15

**Authors:** Farooq M Almutairi

**Affiliations:** 1 Clinical Laboratories Sciences, College of Applied Medical Sciences, University of Hafr Al-Batin, Hafr Al-Batin, SAU

**Keywords:** clinical chemistry, biochemistry, systematic review, c-reactive protein, anastomosis, biomarkers, colorectal

## Abstract

Due to its diverse presentation, anastomotic leakage (AL) following colorectal surgery is challenging to detect and frequently discovered when the patient becomes critically ill. When diagnosing AL in its early stages, biomarkers play a large role. This review was conducted to evaluate the diagnostic value of biomarkers in AL after colorectal surgeries. A literature search was undertaken electronically in major search engines such as Medline (PubMed), Google Scholar, ScienceDirect, EMBASE, and CENTRAL (Cochrane Library) databases. Observational studies of both retrospective and prospective nature were included. Origin Pro 2022 (Origin Labs) software was used to assess the prevalence of AL and generate the forest plot. A total of 13 articles fulfilled the eligibility criteria. A pooled prevalence of 9.19% was noted for AL in colorectal surgeries. In the present review, the observed sensitivity for C-reactive protein (CRP) was 80.5% and the specificity was 84% (postoperative day three). In contrast, these were 100% and 83.9% for procalcitonin on postoperative day five. CRP showed the highest diagnostic accuracy and excels at eliminating AL, but combining biomarkers can increase the diagnostic precision of early detection of AL.

## Introduction and background

One of the most serious side effects following colorectal surgeries is anastomotic leakage (AL), which is linked to increased rates of death and morbidity as well as longer hospital stays [1-4]. It may endanger the prognosis as an independent and confusing factor. AL is still a major problem with no known cause and, in certain situations, no identified risk factors, even with advances in surgical techniques and knowledge of the risk factors [5]. To lower associated morbidity and mortality, anastomotic leaks should be found as soon as possible [6].

Surgeons predict AL poorly when performing abdominal surgery [7]. Routine imaging has the drawback of radiation exposure to the user and is neither reliable nor economical for detecting leaks. A biomarker would therefore be advantageous if it is affordable and sensitive enough to permit the patient’s safe discharge.

A characteristic that can be used to objectively identify the pathogenesis of a disease is called a biomarker. It has been demonstrated in the literature that several biomarkers involved in the healing process following colorectal surgery may be able to identify different stages of early ischemia, inflammation, and necrosis [8]. Currently, the majority of researchers have focused on C-reactive protein (CRP). In addition to being a general indicator of poor surgical and non-surgical outcomes [9], CRP has also been used to diagnose intra-abdominal surgical infections and even to predict survival after liver metastasis resection. Furthermore, research has examined CRP as a potential early indicator of septic issues after pancreatic, rectal, and esophageal resections. In the context of fast-track surgery, an inflammatory measure with a strong negative predictive value might permit safe early hospital discharge. The current review was conducted to evaluate the diagnostic utility of biomarkers in patients presenting with AL following colorectal surgery.

## Review

Methodology

We searched all databases for studies done during the last 15 years (2010-2022), including Medline (PubMed), Google Scholar, ScienceDirect, EMBASE, and CENTRAL (Cochrane Library). Additionally, we looked through online trial records via the ClinicalTrials.gov and www.controlled-trials.com indexes.

Derivatives of “biomarkers,” “markers,” “inflammatory markers,” “anastomotic leaks,” “colorectal surgeries,” “rectal surgeries,” or “surgery” were among the search terms used. For the EMBASE database, comparable search parameters for the title and abstract were applied. We looked through the Cochrane Library index. The linked articles’ references were manually searched. After being compiled from relevant sources, government and legislative reports were manually searched. Every publication examining peritoneal and systemic biomarkers related to anastomotic leaks after colorectal surgery was included in this analysis.

Qualifications for Eligibility

The articles that met the eligibility criteria had to use any biomarkers or inflammatory markers and detail anastomotic leaks after colorectal surgeries. Studies that were no older than 2007 and that included information from the previous 15 years about the relationship between biomarkers and anastomotic leaks were considered for inclusion. The analysis excluded case reports, surgery techniques, and review articles. The restriction was made to only allow English-language articles.

Data Extraction

A standard extraction form was used to compile the data. We collected data on the author, publication year, study design, inclusion period, the total number of patients, type of biomarker used, age, sex, cut-off value, postoperative day, sensitivity, specificity, etc. for each included paper. Patient characteristics from studies that provided adequate specific data were collected, including age and gender. The weighted average by study size was used to compute the mean.

Quality Evaluation

The Newcastle-Ottawa Quality Assessment Scale (cohort studies) was used by the authors to evaluate the studies’ quality when appropriate independently. This assessed the possibility of bias and posed issues with the application. Comparability, outcome, and three selections were used to evaluate each selection individually for risk of bias.

Results

**Figure 1:**
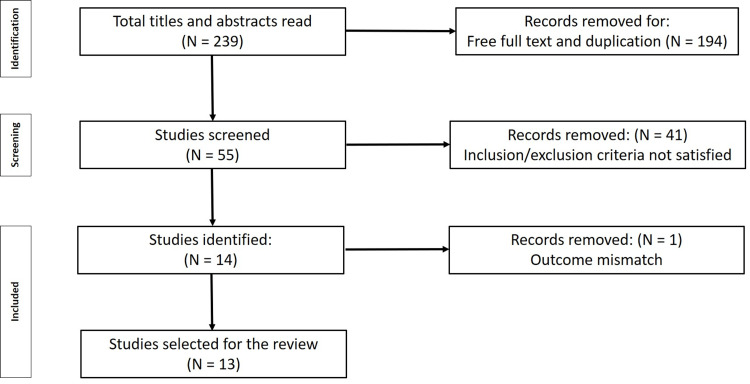
Preferred Reporting Items for Systematic Reviews and Meta-Analyses flowchart of the study.

Following thorough database searches, 13 studies were included in this meta-analysis and qualitative systematic review (Figure 1).

A comparative summary of the included studies is shown in Table 1.

**Table 1:** Comparative summary of included studies. CRP = C-reactive protein; PCT = procalcitonin; WBC = white blood cell count; IL = interleukin; TNF-α = tumor necrosis factor-alpha

Study ID	Sample size	Demographics	Clinical symptoms	Biomarkers assessed	Assessment tool	Study design
Essa et al. (2021) [10]	130	Mean age = 51.4 years; M:F = 73:57	Fever, tachycardia, pain, peritonitis, purulent, or intestinal content in the drain	CRP, WCC, PCT	CRP: Immunonephelometry on an automated Dimension Vista analyzer PCT was measured by the electrochemiluminescence immunoassay Elecsys BRAHMS PCT	Prospective
Almeida et al. (2011) [11]	173	Mean age = 69.5 years; 54.2% vs 45.5%	Peritonitis and free fecal fluid flowing from the drain site or within the abdomen	CRP	Not mentioned	Prospective
Ellebæk et al. (2014) [12]	50	Mean age = 61.5 years	Not mentioned	IL-1, IL-4, IL-6, IL-8, IL-10	Bio-Plex suspension array system	Prospective
Garcia-Granero et al. (2013) [13]	205	Mean age = 63.3 ± 15.5 years; 112 males versus 93 females	Drainage from the wound margin and signs of inflammation	CRP, PCT	CRP serum levels were assessed by the latex immunoturbidimetric method. PCT was measured by electrochemiluminescence immunoassay	Prospective
Giaccaglia et al. (2014) [14]	99	Mean age = 68 years	Peritonitis at the drain site, and the presence of air or fluid in the infected region	CRP, PCT	Dimension Vista analysis	Prospective
Giaccaglia et al. (2016) [15]	504	Mean age = 58.3; 58.3% males and 47.1% females	Wound margins presenting with purulent drainage	PCT, CRP	Not mentioned	Multicentric prospective observational
Käser et al. (2014) [16]	1,106	Mean age = 62.3 years; 67% males, 33% females	Abscess in proximity to the anastomosis	Hyponatremia and leukocytosis	Serum analysis	Prospective
Kørner et al. (2009) [17]	246	Median age = 71 years; males 46% and females 54%	Elevated temperature levels >38°C; inflammatory biochemical markers	CRP	Hematological	Prospective
Wani et al. (2020) [18]	60	Age range = 25 to 70 years	>38°C in 36.7%, absence of bowel action in 23.3%, diarrhea in 73.3%, and renal failure in 16.7%	CRP, PCT, IL-6, IL-10, TNF-α	Not mentioned	Prospective
Ortega-Deballon et al. (2010) [19]	133	Mean age = 65 ± 16 years; M:F ratio = 85:48	Fever, abnormal bowel movements, and abdominal signs	CRP, WBC	Immunonephelometry assay	Prospective
Platt et al. (2012) [20]	454	Age not mentioned specifically; M:F ratio was 62:42	Confirmed radiographically or by relaparotomy	CRP	Turbidimetric assay	Retrospective
Warschkow et al. (2011) [21]	1238	Mean age = 68.2 ± 12.1 years		CRP, WBC	Automated analytic analyzer for CRP	Retrospective
Welsh et al. (2007) [22]	383	Median age = 65 years	Abscess or wound infection at the surgical site	CRP	Turbidometry	Retrospective

The study by Warschkow et al. [21] had the largest sample size of all the included studies, and the study by Wani et al. had the highest percentage of events (anastomotic leaks) [22]. The mean age was 63.85 years based on data from 10 studies that included lifespan. Three of the 13 included studies were retrospective observational, and 10 were prospective. Eleven out of 13 studies (including CRP) compared it to other biomarkers. White blood cell (WBC) was included in four studies, procalcitonin (PCT) was included in five studies, and interleukins were included in two studies (Table 2).

**Table 2:** Comparison of included studies evaluating systemic biomarkers. CRP = C-reactive protein; PCT = procalcitonin; WBC = white blood cell count; IL = interleukin; TNF-α = tumor necrosis factor-alpha; INF = interferon; TLC = total leukocyte count; POD = postoperative day

Authors	Total AL	Biomarkers cut-off	POD	Sensitivity	Specificity
Essa et al. (2021) [10]	10 (7.69%)	CRP = 39.7 ± 7.5 mg/L	3	90%	100%
		WCC = 8.4 ± 1.1	3	90%	72%
		PCT = 2.05 ± 0.21 ng/mL	5	100%	84%
Almeida et al. (2011) [11]	24 (13.8%)	CRP = >70 mg/L	3	92%	9%
Ellebæk et al. (2014) [12]	4 (8%)	Mannan-binding protein	5	NA	NA
		s-MAC, INF-c, and TNF-a = No significant change			
		IL-1ß = 3.77 pg/mL (↓)	5		
		IL-4 = 10.8 pg/mL (↑)	5		
		IL-5 = 0.91 pg/mL (↓)	5		
		IL-6 = 170.6 pg/mL (↑)	5		
		IL-8 = 143 pg/mL (↑)	5		
		IL-10 = 20.9 pg/mL (↑)	5		
Giaccaglia et al. (2014) [14]	17 (8.30%)	CRP = 178.7 mg/L	5	73%	83%
		PCT = 5.24 mg/L	5	100%	72%
	7 (7.10%)	CRP = 22 mg/dL	5	NA	NA
		PCT = 3.17 ng/ml	5	NA	95.70%
Giaccaglia et al. (2016) [15]	28 (5.6%)	CRP = 22 mg/dL	3	73.90%	86%
		PCT = 4.1 ng/ml	3	69.60%	96%
Käser et al. (2014) [16]	81 (7.3%)	Hyponatremia	5	23%	93%
		Leukocytosis	5	58%	79%
Kørner et al. (2009) [17]	18 (7.31%)	CRP = 257 U/mL	3	82%	73%
		WBC = 14,200	3	69%	82%
Wani et al. (2020) [18]	16 (26.70%)	IL-6 = 3101-6330 pg/mL	3	55.80%	100%
		IL-10 = 901-1540 pg/mL	3	82.70%	75%
		INF-α = 19-30 pg/mL	3	84.60%	87.50%
		CRP = 2.2-3.0 mg/L	3	53.80%	12.50%
		TLC = No difference (non-significant)	3	89.10%	12.50%
		PCT = 0.5-1 ng/mL	3	80%	4%
Ortega-Deballon et al. (2010) [19]	21 (15.80%)	CRP = 125 mg/L	4	81.80%	64.40%
		WBC = 10,839 count/mm^3^	4	NA	NA
Platt et al. (2012) [20]	26 (5.72%)	CRP = 190 mg/L	3	77%	80%
Warschkow et al. (2011) [21]	91 (7.80%)	CRP = 123 mg/L	4	66%	77%
		WBC = 8.9 × 10^3^ count/mm^3^	2	49%	64%
Welsch et al. (2007) [22]	22 (5.70%)	CRP = 140 mg/L	3	80%	81%

AL was found to have a pooled prevalence of 9.19% of the sample size. The study by Wani et al. reported the highest prevalence (26.7%), while the study by Giaccaglia et al. reported the lowest prevalence (5.6%) [15].

Cut-Off Point

For CRP on postoperative day (POD) three, the pooled average cut-off value was 45.7 mg/L, and on POD four, it was 124 mg/L. On POD three, the pooled average PCT cut-off value was 2.55 ng/mL, and on POD five, it was 3.48 ng/mL.

Critical Reactivity Parameters (CRP)

On POD three, the pooled average CRP sensitivity was 80.5%, and the specificity was 84%. On POD four, these values were 73.9% and 70.7%, respectively. According to one study, the percentages were 73% and 83%, respectively, on POD five.

Sensitivity and Specificity (PCT)

**Figure 2:**
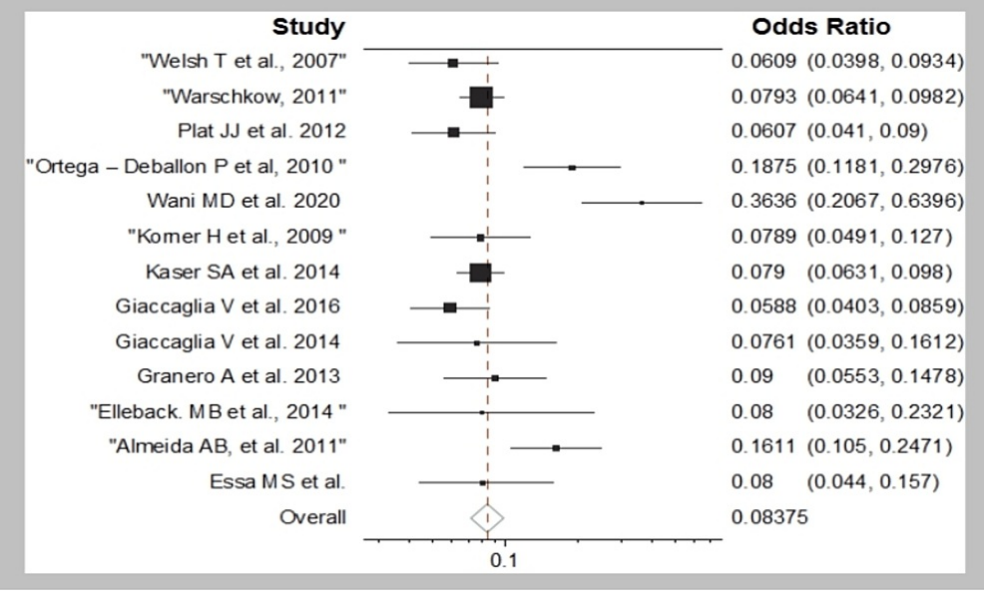
Forest plot showing events/sample. Essa et al. (2021) [10]; Almeida et al. (2011) [11]; Ellebæk et al. (2014) [12]; Garcia-Granero et al. (2013) [13]; Giaccaglia et al. (2014) [14]; Giaccaglia et al. (2016) [15]; Käser et al. (2014) [16]; Kørner et al. (2009) [17]; Wani et al. (2020) [18]; Ortega-Deballon et al. (2010) [19]; Platt et al. (2012) [20]; Welsch et al. (2007) [22].

On POD three, the pooled average PCT sensitivity was 74.8%, while the specificity was 50%. On POD five, on the other hand, the values were 100% and 83.9%, respectively. The prevalence of 0.08 anastomotic leaks is depicted in Figure 2.

The study by Wani et al. [18] reported the highest value (0.3636), whereas the study by Giaccaglia et al. (2016) reported the lowest value (0.058) [15]. The size of the square varied according to the sample size of the study.

The quality of individual studies was evaluated using the Newcastle-Ottawa Quality Assessment Scale for cohort studies (Table 3).

**Table 3:** Newcastle-Ottawa Quality Assessment Scale (cohort studies) Thresholds for converting the Newcastle-Ottawa scales to AHRQ standards (good, fair, and poor): Good quality: Three or four stars in the selection domain AND one or two stars in the comparability domain AND two or three stars in the outcome/exposure domain. Fair quality: Two stars in the selection domain AND one or two stars in the comparability domain AND two or three stars in the outcome/exposure domain. Poor quality: Zero or one star in the selection domain OR zero stars in the comparability domain OR zero or one star in the outcome/exposure domain.

Studies	Categories		
	Selection	Comparability	Outcome
Essa et al. (2021) [10]	****	**	**
Almeida et al. (2011) [11]	****	-	**
Ellebæk et al. (2014) [12]	****	*	***
Garcia-Granero et al. (2013) [13]	****	**	***
Giaccaglia et al. (2014) [14]	****	**	**
Giaccaglia et al. (2016) [15]	****	**	***
Käser et al. (2014) [16]	****	**	***
Kørner et al. (2009) [17]	****	**	***
Wani et al. (2020) [18]	****	**	**
Ortega-Deballon et al. (2010) [19]	****	**	***
Platt et al. (2012) [20]	****	-	***
Warschkow et al. (2011) [21]	****	**	***
Welsch et al. (2007) [22]	****	-	***

Three criteria were used to evaluate each study individually, i.e., selection, comparability, and outcome. Six studies out of the 13 studies were given a perfect maximum of nine stars, indicating good quality. In the quality assessment, eight studies were awarded eight stars, two were awarded seven stars, and only one study was awarded six stars. Nine studies at most received perfect scores (2/2 stars) in the comparability category and perfect (3/3 stars) in the outcome category, out of all the studies that received perfect scores (4/4 stars) in the selection categories.

Discussion

This review aimed to provide a general summary of how well biomarkers predict AL after colorectal surgeries. With multiple time points and varied cut-off values, this systematic review found various systemic biomarkers that were significantly enhanced in the presence of AL. When evaluated individually, the biomarkers generally demonstrated subpar diagnostic accuracy in predicting AL, but when combined, the accuracy was enhanced.

The specific pathophysiology of AL is still largely understood, despite substantial research using animal models and human investigations [23,24]. The identification of novel leads for biomarkers or therapeutics is hampered by this knowledge gap [25]. Early or late postoperative development of AL is generally seen, and it is thought that each of these outcomes results from distinct pathophysiologic processes [26]. An early leak is more likely to be due to a technical flaw, whereas a late leak may be the result of an increased oral intake after discharge or an early clinically occult leak [27,28]. Both early and late leaks need to be identified or anticipated as soon as feasible, preferably using a minimally intrusive objective method, regardless of the chronology or etiology.

Regarding to biomarkers, leucocytes and CRP are often observed, which are acute-phase reactants, and are increased when an inflammatory response occurs due to both viral and non-infectious causes [29]. An increased CRP can be a sign of a postoperative infectious complication, especially on PODs three and four, when the inflammatory response of the resection has subsided in patients with no problems [17,30,31,32]. However, because CRP is increased in both medical and surgical situations, it cannot accurately distinguish between the two [33,34]. Instead, because it has a useful negative predictive value and can avoid the need for potentially damaging swallow investigations, its strength lies in excluding AL on PODs three to five [27,35-38]. Leucocytes are biomarkers for AL but, similar to CRP, are more accurate when they exclude rather than indicate this postoperative complication [33,38,39].

In this study, we found that CRP can be an indicator of AL with a rise during PODs three to five, having sensitivity and specificity above 80%, and is an overall negative predictive test. Similar conclusions were reached by Aolfi et al. in a recently published systematic review and meta-analysis, who also found that CRP can be a useful marker to rule out leakage with encouraging clinical and radiographic indications [40]. Supported by the findings of Singh et al. and Haga et al., it is a helpful negative predictive test but not a very reliable positive predictor of the emergence of AL [41,42].

PCT is thought to be a more accurate indicator of severe infections and sequelae compared to CRP and leucocytes [13,43-45]. Increased PCT levels may be a specific sign of a combination of surgical and infectious complications, among which AL is the most frequent [46]. Overall findings have been mixed, and it is still unclear if PCT can distinguish between the various postoperative complication subtypes [39,44,47]. Additionally, PCT is more expensive than CRP or leukocytes and is not typically used in laboratory tests [43,48]. However, some studies report that PCT is a useful negative test for AL following elective colorectal surgery and, as an isolated test, it is not useful in detecting AL [49].

According to the findings of this comprehensive research, no one biomarker can definitively detect or forecast AL. As it cannot consistently distinguish between surgical and infectious sequelae, CRP, which has the highest diagnostic accuracy, excels at eliminating AL. To predict or identify AL, however, until a new, more precise biomarker is discovered, a synergistic effect generated by combining other biomarkers with strong diagnostic accuracies, such as CRP, PCT, and amylase, should be used.

Being a single-author research, the chances of possible biases in the selected studies cannot be ruled out.

## Conclusions

After colorectal resection surgery, the early diagnosis of AL is aided by several distinct biomarkers. Due to their low sensitivity and positive predictive value, these biomarkers are generally poor predictors of AL. However, due to the wide variety of cut-off values and PODs involved, various diagnostic accuracies have been discovered. Combining biomarkers can increase the diagnostic precision of early detection of AL. Current biomarkers help differentiate between patients at low risk for AL and those at high risk who can benefit from further imaging.

To find a near-perfect biomarker that reflects the perianastomotic environment for predicting AL in the early postoperative period before discharge, high-quality prospective trials with precise definitions of AL are required.
